# β-Cell Failure or β-Cell Abuse?

**DOI:** 10.3389/fendo.2018.00532

**Published:** 2018-09-13

**Authors:** Karel Erion, Barbara E. Corkey

**Affiliations:** ^1^Division of Endocrinology, Department of Medicine, David Geffen School of Medicine, University of California, Los Angeles, Los Angeles, CA, United States; ^2^Evans Department of Medicine, Obesity Research Center, Boston University School of Medicine, Boston, MA, United States

**Keywords:** hyperinsuilnemia, beta-cell, obesity, insulin resistance, type 2 diabetes

## Abstract

This review is motivated by the need to question dogma that has not yielded significant improvements in outcomes of Type 2 Diabetes treatment: that insulin resistance is the driver of ß-Cell failure and resulting hyperglycemia. We highlight the fact that hyperlipidemia, insulin resistance, and hyperinsulinemia all precede overt diabetes diagnosis and can each induce the other when tested experimentally. New research highlights the importance of high levels of circulating insulin as both a driver of weight gain and insulin resistance. Data from our lab and others document that several nutrients and environmental toxins can stimulate insulin secretion at non-stimulatory glucose in the absence of insulin resistance. This occurs either by direct action on the ß-Cell or by shifting its sensitivity to known secretagogues. We raise the next logical question of whether ß-Cell dysfunction in Type 2 Diabetes is due to impaired function, defined as failure, or if chronic overstimulation of the ß-Cell that exceeds its capacity to synthesize and secrete insulin, defined as abuse, is the main abnormality in Type 2 Diabetes. These questions are important as they have direct implications for how to best prevent and treat Type 2 Diabetes.

## Metabolic abnormalities preceding type 2 diabetes (T2D)

A long prodrome precedes the diagnosis of T2D that includes elevated fasting insulin, obesity, insulin resistance (IR), and dyslipidemia. According to the American Diabetes Association, fasting glucose values of 5.5 mM or less are considered normal, values between 5.6 and 6.9 are considered pre-diabetic and values above 7.0 mM define T2D. These are arbitrary thresholds since fasting plasma glucose does not exhibit a bi- or tri-modal distribution that might be used to derive clear cut points for pre-diabetes or diabetes diagnosis (1). Like other components of the prodrome, glucose levels also increase prior to diagnosis.

However, elevations in blood glucose are a late manifestation of a disease that is preceded by other well-established indicators of diminishing metabolic health including obesity with accompanying lipid abnormalities or hyperlipidemia (HL), elevated fasting insulin or hyperinsulinemia (HI), and IR. IR is usually described as the proximal cause of T2D that occurs only when the ß-cell can no longer compensate for the increasing demands of IR (ß-cell decline). However, IR is also defined by elevated insulin levels! Interestingly, there is no established or proposed mechanism by which IR can stimulate secretion except via glucose, which does not change during the so-called “compensatory” stage of the disease (2). Furthermore, there is no evidence in most cases that IR precedes or causes HI. There is also no evidence that HI does not occur first and cause IR (3, 4). Knowledge of what comes first is essential to establish causality and hence optimize preventive treatment if HI is indeed the initiating cause of IR.

There are logical flaws in the dogma attributing T2D to IR and defining T2D as the moment ß-cells cannot keep up with demand. Secretory systems do not possess an infinite capacity to synthesize and secrete hormones but rather have a finite capacity for work with a distinct maximum secretory potential. Stimulation of ß-cells beyond maximum cellular secretory capacity constitutes abuse, whether through continuous excess carbohydrate or lipid exposure or environmental toxins (5–7). Determination of whether T2D results from ß-cell failure or ß-cell abuse is essential to inform appropriate therapeutic interventions. If failure, or inability to respond is the problem, then the solution is to add exogenous insulin or stimulate its secretion further in response to elevated glucose, as is current practice. If ß-cell abuse, due to exceeding the maximum secretory capacity of a normally constituted ß-cell is the problem, then the solution is to stop the abuse as early as possible by diminishing HI, particularly if the abuse ultimately leads to ß-cell failure.

## The evidence on trajectories or sequence of development of metabolic abnormalities in the population

Large longitudinal studies could inform the relationships among HL, IR, and HI, the three key metabolic biomarkers that precede overt T2D. However, available data sets do not appear to provide convincing support for a specific sequence of development. On the other hand, animal studies and some human studies do provide limited evidence that each metabolic abnormality can lead to the other two if sustained, thus underpinning the rationale for seeking such clarification. For example, HL induced by high fat feeding in animals, or lipid infusion in humans, increases insulin secretion and induces IR (8). In another study evaluating the trajectory of metabolic changes, a linear increase in fasting glucose started 3 years before diagnosis of diabetes, whereas insulin sensitivity (HOMA) decreased during the 5 years before and ß-cell function increased 4 years before diagnosis (9). In another study, South Asians exhibited increased fasting insulin and 2-h stimulated insulin well before diagnosis and demonstrated increased ß-cell function (HOMA2- ß) 7 years before diagnosis (10). Preteen HI predicts weight gain and T2D (9). Interestingly, BMI in young Finns (11–14) was not associated with adult T2D but fasting insulin was (15): in assessing parameters that predict T2D, it was found that progressors had high fasting and 2-h insulin levels. These limited data predict that HI can be a cause of both obesity (16) and IR. However, the frequency of specific trajectories in defined populations is not known and further research is needed to document the sequence of appearance of these biomarkers in the progression to T2D in order to identify the appropriate focus for prevention.

**Figure 1:**
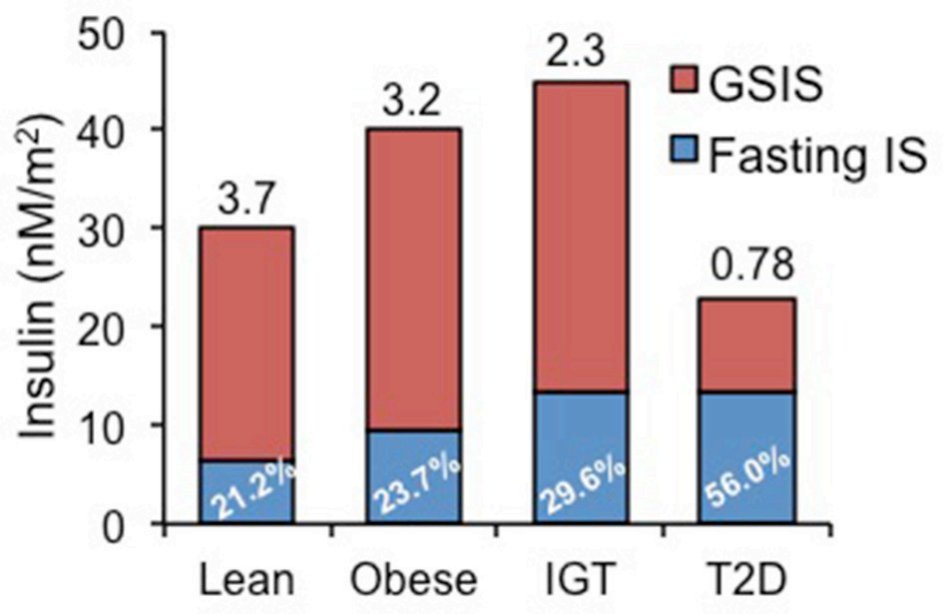
Basal (fasting) and glucose-stimulated insulin secretion (GSIS) rates in lean, obese, patients with impaired glucose tolerance (IGT) and diabetics (T2D). Data redrawn from Ferrannini et al. (17), showing increasing percentage of basal insulin secretion rate relative to total insulin secretion rate and diminishing GSIS in IGT and T2D. Number above each bar represents the ratio of GSIS to basal insulin secretion rate.

The importance of distinguishing basal vs. glucose-stimulated insulin secretion (GSIS) is depicted in Figure 1 [redrawn from Ferrannini et al. (17) based on data from 188 subjects undergoing a 120 min, 75 g oral glucose tolerance test]. In this figure, basal HI begins to manifest in obese individuals and in subjects with impaired glucose tolerance (IGT) as a rising basal and an increasing percentage of total insulin secretion (in blue). The ratio of GSIS to basal (GSIS fold increase shown above bars in Figure 1) is lower in obesity and IGT and is markedly reduced in T2D, although basal HI is sustained. Decreased GSIS, in conjunction with increased HI, was shown by mathematical modeling of ß-cell glucose responses to be the hallmark of T2D progression (17).

## The proinsulin:insulin ratio as a marker for overstimulation of secretion from the ß-cell

Stimulation of the ß-cell during progression toward T2D may even be underestimated, as current assays do not take into account circulating proinsulin. Proinsulin is the immature form of the hormone insulin that is packaged into secretory granules at the Golgi apparatus during granule biogenesis. Cleavage of C-peptide by prohormone convertase enzymes PCSK2 and PCSK3 within the granule results in mature insulin which can then be secreted into circulation upon fusion of the granule with the plasma membrane (18). Under normal conditions conversion of proinsulin to insulin occurs to such an extent that secretion of proinsulin is nominal (19). However, as patients progress toward overt T2D there is a steady increase in proinsulin secretion and the circulating proinsulin:insulin ratio (20–22). Two hypotheses have emerged to account for this phenomenon: (1) reduced activity of prohormone convertase enzymes, possibly due to a rise in pH within the insulin granules or (2) a prolonged increase in the rate of secretion prevents adequate time with which to properly process proinsulin to insulin. It is known that conversion of proinsulin to insulin takes ~3 h (23).

Several factors argue that the increased proinsulin secretion in T2D is due to abuse, or prolonged stimulation of secretion beyond the normal capacity of the ß-cell. First, we have previously published that changes in the secreted proinsulin:insulin ratio can be achieved in cultured ß-cells by chronic exposure to excess lipid (24). This indicates that direct action on the ß-cell and not IR *per se* is responsible for the aberrant proinsulin secretion. Second, artificial elevation of glucose for a prolonged period increases the proinsulin:insulin ratio in the absence of any apparent defect of proinsulin processing (25). Lastly, induction of ß-cell rest either pharmacologically or via bariatric surgery rapidly normalizes the secreted proinsulin:insulin ratio (26, 27). These results even lead to the possibility that ß-cell failure may result from prolonged overstimulation, as the proinsulin:insulin ratio remains significantly elevated in T2D despite the inability to respond acutely to stimuli. Above 7.0 mM glucose, it is reasonable to state that ß-cells are likely to be in a chronically stimulatory state. Additionally, ß-cells exposed to excess nutrients in the form of glucose and fatty acids have a left-shifted dose response for glucose-stimulated insulin secretion, further increasing the drive of the ß-cell to secrete insulin (7).

## Could diabetes be caused by insulin hypersecretion?

This raises the inadequately explored possibility that diabetes could be caused by long-term ß-cell overstimulation that ultimately exceeds the maximum capacity of the secretory pathway. Such a possibility is consistent with the 9-fold increase in fasting, unstimulated insulin levels reported in some obese diabetic subjects (28). In contrast, a 9-fold increase in GSIS is considered a robust response in a lean individual.

ß-Cell hypersecretion of insulin in the absence of a stimulatory fuel increases fat stores (24). HI also can cause IR through insulin-induced receptor down-regulation both in the periphery (29) and in the brain where HI-induced insulin resistance may abrogate its normal role as a satiety signal (30, 31). In addition, insulin causes IR by inducing lipogenesis and increasing lipid metabolites that are known to diminish insulin sensitivity (32).

Thus, HI can precede IR as shown in studies that artificially increased insulin in the circulation in man and rodents to cause IR and weight gain (33–35). Interestingly, inhibition of insulin secretion under HI conditions may not cause hyperglycemia but rather may improve weight loss when combined with dieting in obese humans (36–38). Further support for an initiating role for HI is the ability to predict diabetes in subjects with high plasma insulin concentrations among Pima Indians (39).

Rodents overexpressing the human insulin gene, or treated with exogenous insulin develop IR secondary to HI (33). In contrast, lowering insulin levels with diazoxide increases insulin sensitivity in rodents and humans (36, 37, 40). Lowering insulin using a novel ß-cell K_ATP_ channel opener, NN414, also proved beneficial in rodents and humans (41–46).

**Figure 2:**
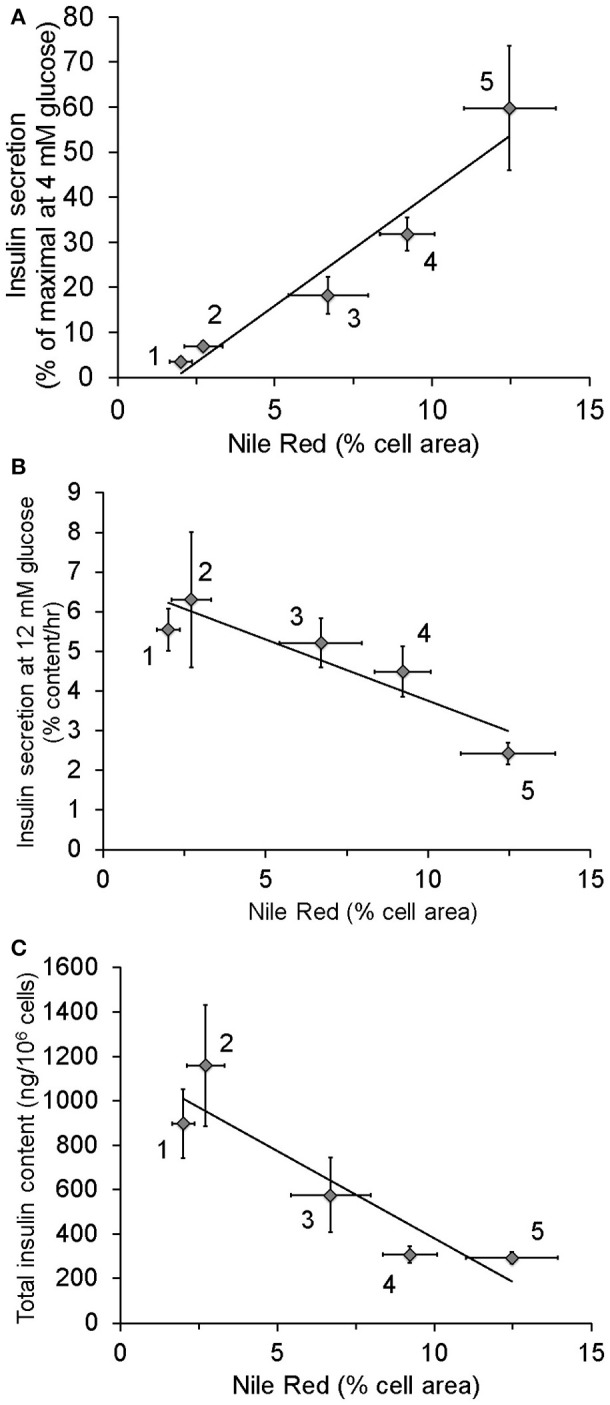
Relationship between lipid stores, insulin secretion, and insulin content in INS1 (832/13) cells cultured in the presence of different nutrients: 1, 4 mM glucose for more than 2 weeks (chronic) (*n* = 12); 2, 4 mM glucose for 48 h (*n* = 8); 3, 4 mM glucose chronic + 0.15 mM oleate for 24 h (*n* = 6); 4, 11 mM glucose (*n* = 13); 5, 11 mM glucose + 0.15 mM oleate for 48 h (*n* = 4). As standard culture media glucose concentration for INS1 cells is 11 mM, cells identified as being cultured in 4 mM glucose were switched from 11 to 4 for the stated period of time. Lipid content was measured using Nile Red. **(A)** Relationship between Insulin secretion at 4 mM glucose following washout of growth media and lipid stores. **(B)** Correlation of intracellular lipid stores and insulin secretion at 12 mM glucose. **(C)** Relationship between lipid stores and total insulin content. Data in this figure were recalculated from previously published results (7).

Data from our laboratory have demonstrated that cultured clonal ß-cells exposed to excess nutrients for as little as 24–48 h exhibit increased lipid content, increased basal insulin secretion, decreased insulin content and diminished GSIS as illustrated in Figure 2. Interestingly, these *in vitro* changes provide a model of what might happen over time *in vivo*. Using this model, we demonstrated that bezafibrate, a pan-PPAR agonist that decreases cellular triglyceride content by stimulating fat oxidation (47), prevents elevated basal insulin secretion. Additionally, we have observed that replacing long-chain fat with medium-chain fatty acid, which is not effectively stored as triglyceride, did not cause hypersecretion (data not shown) unlike long chain fatty acids (7). These findings implicate excess lipid stores in the ß-cell as complicit in the induction of HI.

We and others have also identified environmental agents that can stimulate insulin secretion at basal glucose levels. These include common food additives (5, 28) estrogenic compounds including bis-phenol A (48, 49), plant extracts (50, 51), and even viruses (52).

Studies are needed to determine whether IR is primary, or secondary to HI, in animal models and in pre-diabetic humans treated with inhibitors of insulin secretion or nutrient regimens that markedly decrease the daily insulin requirement. Analysis of available data with diazoxide, NN414, and somatostatin support the concept that inhibition of insulin secretion can improve metabolic health in some cases (41–46).

## Bariatric surgery has led to amazing insights into diabetes

Recovery from ß-cell hypersecretion of insulin and amelioration of T2D occurs most frequently following bariatric surgery, but also less frequently after weight loss or a very low carbohydrate diet, provided that sufficient secretory capacity has been retained by the ß-cells. The majority of patients with T2D are rapidly “cured” following bariatric surgery. Normalization of circulating insulin and glucose levels occurs within a week of surgery without appreciable weight loss (16). In contrast, normalization of muscle insulin sensitivity requires at least 3 months, although it too is eventually achieved (53). The mechanisms explaining this normalization of HI and T2D are unknown. The relatively quick recovery of ß-cell function again implicates hypersecretion as the main driver of ß-cell dysfunction in T2D, as generation of new islet cells is known to be very slow in adult humans (53, 54). Unfortunately, this reset of metabolic parameters is transient in some patients. T2D recurs in a percentage of bariatric surgery patients after several years (55, 56), and more frequently in African Americans. T2D is characterized by HI and that is the abnormality that is reset most rapidly following surgery but is also the earliest to recur in patients in whom T2D returns (16).

The common features of effective treatments are diminished ß-cell demand and markedly decreased HI prior to resolution of IR, decreased intake of simple carbohydrates, decreased calorie intake, and increased fat oxidation. It should be noted that the decrease in HI following bariatric surgery is much greater than that induced by paired nutrient intake (57) implicating a mechanism other than altered nutrient intake.

## Current dogma underpins treatment: problems with the current therapeutic approaches

T2D and its complications continuously worsen over the course of the disease. Initial single drug therapy is followed by additional drugs as the disease and its complications continue to worsen. The first therapeutic given to newly diagnosed patients with T2D or even pre-diabetes is metformin, which does not act on the ß-cell, but rather has been reported to decrease both BMI and HI (58–60), relieving rather than exacerbating some of the stress on the ß-cell. However, metformin is not a cure as the disease generally progresses and requires additional medication to control blood glucose.

Despite the fact that T2D involves many metabolic abnormalities, treatment is focused only on normalizing glucose and is based on the dogma that “IR is the cause of T2D.” This has inhibited investigation into the alternative possibilities that T2D is a consequence of HL or HI and that these metabolic abnormalities precede and cause both IR and hyperglycemia.

In contrast to current dogma, if HI is the cause of IR, then stimulating further insulin secretion to overcome resistance may adversely impact long-term function of the ß-cell, assuming that secretory capacity is not unlimited. If HI is the cause of IR, then stimulating further insulin secretion may actually sustain and worsen IR. Furthermore, HI may cause or increase obesity that will in turn sustain HL, HI, and IR. Decades of failure to prevent or reverse the diabetes epidemic based on the “IR dogma” suggest that the time has come to challenge this dogma and investigate other possibilities.

## Other possibilities. could IR, obesity, and diabetes be caused by aberrant lipid signaling or environmental chemicals?

This perspective has focused, up to now, on HI as a potential initiating defect. An alternative and equally compelling cause of T2D could be excessive lipid accumulation and signaling in metabolically sensitive tissues. There is an association with lipid abnormalities in both HI and IR but where this fits into the sequence of deterioration has not been established. Evidence has been obtained that excess lipid accumulation increases basal insulin secretion (Figure 2), and induces IR in muscle and in liver (61–63). It is well established that there is a strong link between lipid abnormalities, particularly in diabetes and cardiovascular disease but here too the cause and effect relationships are still uncertain.

Importantly, HL is associated with diminished insulin clearance by the liver, an important contributor to HI (64, 65) and may be a compensatory mechanism in response to excessive ß-cell stimulation. In insulin resistant states both HI and decreased insulin degradation occur together.

Recovery from ectopic lipid mediated HI and IR has also been shown to occur post gastric surgery, following weight loss or in response to a ketogenic diet. Assessment of whether HL is primary or secondary to IR or HI may be determined in animal models and pre-diabetic humans using stimulators of PPARα. Review of published studies with fibrates show a correlation between triglyceride levels and HbA1c in diabetic patients and show that bezafibrate improves HbA1c in patients with diabetes (66–70).

An additional potentially major contributor to obesity, diabetes and/or ß-cell stress that requires serious consideration and much more research is the role of environmental chemicals. Evidence exists for an association of some persistent-organic-pollutants (POPs) with T2D but not enough to establish causality (71). Similarly, there is suggestive evidence for a role of arsenic in T2D (72). In addition, there is a positive association between obesity in offspring and maternal smoking during pregnancy (73) although further studies are needed to assess the link to T2D, if any. Confirmation of a causal association between environmental toxins and metabolic dysfunction also will require determining the targets of such chemicals or the removal of such agents from the environment. The temporal relationship, if any, between environmental toxins and either HI, HL, or IR is not known but could provide important insight into the initiating and sequential metabolic abnormalities leading to T2D. Current advancements in analytical techniques may permit more widespread assessment of the relationship between blood levels of environmental toxins and metabolic dysfunction.

## Conclusions: ß-cell failure or ß-cell abuse?

To answer this question, we must determine whether diabetes is a disease of limited glucose storage capacity or an adaptive response to excess lipid or a toxic environment. It seems unlikely that unlimited capacity to secrete insulin is required of a healthy ß-cell. However, by the time the disease is manifested, diabetics may be diminished in their capacity to handle glucose and could benefit from ß-cell rest by initiating a more rigorous effort to decrease simple carbohydrate intake before ß-cell failure worsens. Luckily unlike certain amino acids and fatty acids, carbohydrates are not an essential component of any diet and can be readily produced endogenously by the liver. Finally, we do not know whether inhibiting HI in normoglycemic individuals could be beneficial or would have adverse effects on glucose homeostasis.

The recognition of our failure to prevent or reverse diabetes, despite concerted research investigations, suggests that it is imperative to rigorously assess alternatives to current dogma. Obesity/T2D may be the biggest epidemic in human history (74). To prevent T2D, a better understanding of the drivers of this epidemic is needed. There has been comprehensive attention to genes, lifestyle and behavior, and current attention on the impact of the intra-uterine environment, epigenetics and the microbiome. None of this has yet proved beneficial in treating or preventing T2D.

Metabolic disease is increasing, and is chronic, expensive and debilitating. If current therapy is contributing to the problem, correction is needed. There is no evidence that ß-cell failure is due to defective performance of ß-cells. It is equally plausible that excess stress and overwork exceed a capacity that would be perfectly functional if the stress were removed. The capacity to drastically improve ß-cell function in Type 2 Diabetic patients soon after bariatric surgery best illustrates this point. If correct, the optimal early intervention might be removal of stress and inhibition of fasting HI. It is also possible that altered lipid metabolism is the cause of HI, IR, and decreased insulin clearance. In this case, decreasing ectopic lipid might be the solution. On the other hand, if induction of metabolic dysfunction by environmental toxins is responsible, the solution would be to identify and remove such toxins.

Stimulation of GSIS when the normal capacity to respond is lost, i.e., following diagnosis of disease may never be an appropriate intervention. Thus, incorrect interpretation of causation may increase stress, damage ß-cells and speed the onset of permanent insulin insufficiency. Large-scale human studies are needed to unequivocally demonstrate the benefit, or lack thereof, of reduced HI in T2D prevention or treatment. It will be critical to determine whether certain populations may benefit more than others in response to a reduction in HI.

## Author contributions

All authors listed have made a substantial, direct and intellectual contribution to the work, and approved it for publication.

### Conflict of interest statement

The authors declare that the research was conducted in the absence of any commercial or financial relationships that could be construed as a potential conflict of interest. The handling Editor declared a past co-authorship with one of the authors (BC).

## References

[1] Vistisen D Colagiuri S Borch-Johnsen K Collaboration D. Bimodal distribution of glucose is not universally useful for diagnosing diabetes. Diabetes Care (2009) 32:397–403. 10.2337/dc08-0867 19074990 PMC2646016

[2] Polonsky KS Given BD Van Cauter E. Twenty-four-hour profiles and pulsatile patterns of insulin secretion in normal and obese subjects. J Clin Invest. (1988) 81:442–8. 10.1172/JCI113339 3276730 PMC329589

[3] Blackard WG Guzelian PS Small ME. Down regulation of insulin receptors in primary cultures of adult rat hepatocytes in monolayer. Endocrinology (1978) 103:548–53. 10.1210/endo-103-2-548 744100

[4] Freychet P Forgue E Le Marchand Y Laudat MH. [Decreased number of insulin receptors in obesity: studies in the obese hyperglycemic mouse (author's transl)]. Ann D'endocrinol. (1976) 37:87–8. 1008511

[5] Simmons AL Schlezinger JJ Corkey BE. What are we putting in our food that is making us fat? Food additives, contaminants, and other putative contributors to obesity. Curr Obes Rep. (2014) 3:273–85. 10.1007/s13679-014-0094-y 25045594 PMC4101898

[6] Berdan CA Erion KA Burritt NE Corkey BE Deeney JT. Inhibition of monoacylglycerol lipase activity decreases glucose-stimulated insulin secretion in INS-1 (832/13) cells and rat islets. PLoS ONE (2016) 11:e0149008. 10.1371/journal.pone.0149008 26867016 PMC4750965

[7] Erion KA Berdan CA Burritt NE Corkey BE Deeney JT. Chronic exposure to excess nutrients left-shifts the concentration dependence of glucose-stimulated insulin secretion in pancreatic beta-cells. J Biol Chem. (2015) 290:16191–201. 10.1074/jbc.M114.620351 25934392 PMC4481219

[8] Boden G Chen X. Effects of fat on glucose uptake and utilization in patients with non-insulin-dependent diabetes. J Clin Invest. (1995) 96:1261–8. 10.1172/JCI118160 7657800 PMC185747

[9] Tabak AG Jokela M Akbaraly TN Brunner EJ Kivimaki M Witte DR. Trajectories of glycaemia, insulin sensitivity, and insulin secretion before diagnosis of type 2 diabetes: an analysis from the Whitehall II study. Lancet (2009) 373:2215–21. 10.1016/S0140-6736(09)60619-X 19515410 PMC2726723

[10] Hulman A Simmons RK Brunner EJ Witte DR Faerch K Vistisen D. Trajectories of glycaemia, insulin sensitivity and insulin secretion in South Asian and white individuals before diagnosis of type 2 diabetes: a longitudinal analysis from the Whitehall II cohort study. Diabetologia (2017) 60:1252–60. 10.1007/s00125-017-4275-6 28409212 PMC5487604

[11] Tirosh A Shai I Afek A Dubnov-Raz G Ayalon N Gordon B. Adolescent BMI trajectory and risk of diabetes versus coronary disease. N Engl J Med. (2011) 364:1315–25. 10.1056/NEJMoa1006992 21470009 PMC4939259

[12] Juonala M Magnussen CG Berenson GS Venn A Burns TL Sabin MA. Childhood adiposity, adult adiposity, and cardiovascular risk factors. N Engl J Med. (2011) 365:1876–85. 10.1056/NEJMoa1010112 22087679

[13] Yeung EH Zhang C Louis GM Willett WC Hu FB. Childhood size and life course weight characteristics in association with the risk of incident type 2 diabetes. Diabetes Care (2010) 33:1364–9. 10.2337/dc10-0100 20215459 PMC2875455

[14] Bell LM Byrne S Thompson A Ratnam N Blair E Bulsara M. Increasing body mass index z-score is continuously associated with complications of overweight in children, even in the healthy weight range. J Clin Endocrinol Metab. (2007) 92:517–22. 10.1210/jc.2006-1714 17105842

[15] Sabin MA Magnussen CG Juonala M Shield JP Kahonen M Lehtimaki T. Insulin and BMI as predictors of adult type 2 diabetes mellitus. Pediatrics (2015) 135:e144–51. 10.1542/peds.2014-1534 25535261

[16] Kelly CT Mansoor J Dohm GL Chapman WH III Pender JRT Pories WJ. Hyperinsulinemic syndrome: the metabolic syndrome is broader than you think. Surgery (2014) 156:405–11. 10.1016/j.surg.2014.04.028 24962189

[17] Ferrannini E Gastaldelli A Miyazaki Y Matsuda M Mari A DeFronzo RA. beta-Cell function in subjects spanning the range from normal glucose tolerance to overt diabetes: a new analysis. J Clin Endocrinol Metab. (2005) 90:493–500. 10.1210/jc.2004-1133 15483086

[18] Kaufmann JE Irminger JC Mungall J Halban PA Proinsulin conversion in GH3 cells after coexpression of human proinsulin with the endoproteases PC2 and/or PC3. Diabetes (1997) 46:978–82. 10.2337/diab.46.6.978 9166668

[19] Horwitz DL Starr JI Mako ME Blackard WG Rubenstein AH. Proinsulin, insulin, and C-peptide concentrations in human portal and peripheral blood. J Clin Invest. (1975) 55:1278–83. 10.1172/JCI108047 1133173 PMC301883

[20] Vangipurapu J Stancakova A Kuulasmaa T Kuusisto J Laakso M. Both fasting and glucose-stimulated proinsulin levels predict hyperglycemia and incident type 2 diabetes: a population-based study of 9,396 Finnish men. PLoS ONE (2015) 10:e0124028. 10.1371/journal.pone.0124028 25853252 PMC4390238

[21] Yoshioka N Kuzuya T Matsuda A Taniguchi M Iwamoto Y. Serum proinsulin levels at fasting and after oral glucose load in patients with type 2 (non-insulin-dependent) diabetes mellitus. Diabetologia (1988) 31:355–60. 10.1007/BF02341503 3046976

[22] Li M Feng D Zhang K Gao S Lu J. Disproportionately elevated proinsulin levels as an early indicator of beta-cell dysfunction in nondiabetic offspring of Chinese diabetic patients. Int J Endocrinol. (2016) 2016:4740678. 10.1155/2016/4740678 27746815 PMC5055967

[23] Sizonenko S Irminger JC Buhler L Deng S Morel P Halban PA. Kinetics of proinsulin conversion in human islets. Diabetes (1993) 42:933–6. 10.2337/diab.42.6.933 8495816

[24] Erion KA Corkey BE. Hyperinsulinemia: a cause of obesity? Curr Obes Rep. (2017) 6:178–86. 10.1007/s13679-017-0261-z 28466412 PMC5487935

[25] Alarcon C Leahy JL Schuppin GT Rhodes CJ. Increased secretory demand rather than a defect in the proinsulin conversion mechanism causes hyperproinsulinemia in a glucose-infusion rat model of non-insulin-dependent diabetes mellitus. J Clin Invest. (1995) 95:1032–9. 10.1172/JCI117748 7883951 PMC441437

[26] Laedtke T Kjems L Porksen N Schmitz O Veldhuis J Kao PC. Overnight inhibition of insulin secretion restores pulsatility and proinsulin/insulin ratio in type 2 diabetes. Am J Physiol Endocrinol Metab. (2000) 279:E520–8. 10.1152/ajpendo.2000.279.3.E520 10950818

[27] Purnell JQ Johnson GS Wahed AS Dalla Man C Piccinini F Cobelli C. Prospective evaluation of insulin and incretin dynamics in obese adults with and without diabetes for 2 years after Roux-en-Y gastric bypass. Diabetologia (2018) 61:1142–54. 10.1007/s00125-018-4553-y 29428999 PMC6634312

[28] Corkey BE. Diabetes: have we got it all wrong? Insulin hypersecretion and food additives: cause of obesity and diabetes? Diabetes Care (2012) 35:2432–7. 10.2337/dc12-0825 23173132 PMC3507569

[29] Gavin JR III Roth J Neville DM Jr de Meyts P Buell DN. Insulin-dependent regulation of insulin receptor concentrations: a direct demonstration in cell culture. Proc Natl Acad Sci USA. (1974) 71:84–8. 10.1073/pnas.71.1.84 4359334 PMC387937

[30] Schwartz MW Marks JL Sipols AJ Baskin DG Woods SC Kahn SE. Central insulin administration reduces neuropeptide Y mRNA expression in the arcuate nucleus of food-deprived lean (Fa/Fa) but not obese (fa/fa) Zucker rats. Endocrinology (1991) 128:2645–7. 10.1210/endo-128-5-2645 2019270

[31] Chavez M Kaiyala K Madden LJ Schwartz MW Woods SC. Intraventricular insulin and the level of maintained body weight in rats. Behav Neurosci. (1995) 109:528–31. 10.1037/0735-7044.109.3.528 7662162

[32] Liu HY Hong T Wen GB Han J Zuo D Liu Z. Increased basal level of Akt-dependent insulin signaling may be responsible for the development of insulin resistance. Am J Physiol Endocrinol Metab. (2009) 297:E898–906. 10.1152/ajpendo.00374.2009 19638508 PMC2763787

[33] Destefano MB Stern JS Castonguay TW. Effect of chronic insulin administration on food intake and body weight in rats. Physiol Behav. (1991) 50:801–6. 10.1016/0031-9384(91)90021-F 1685590

[34] Larue-Achagiotis C Goubern M Laury MC. Concomitant food intake and adipose tissue responses under chronic insulin infusion in rats. Physiol Behav. (1988) 44:95–100. 10.1016/0031-9384(88)90351-4 3070583

[35] Del Prato S Leonetti F Simonson DC Sheehan P Matsuda M DeFronzo RA. Effect of sustained physiologic hyperinsulinaemia and hyperglycaemia on insulin secretion and insulin sensitivity in man. Diabetologia (1994) 37:1025–35. 10.1007/BF00400466 7851681

[36] Alemzadeh R Jacobs W Pitukcheewanont P. Antiobesity effect of diazoxide in obese Zucker rats. Metabolism (1996) 45:334–41. 10.1016/S0026-0495(96)90287-5 8606640

[37] Alemzadeh R Langley G Upchurch L Smith P Slonim AE. Beneficial effect of diazoxide in obese hyperinsulinemic adults. J Clin Endocrinol Metab. (1998) 83:1911–5. 10.1210/jc.83.6.1911 9626118

[38] Greenwood RH Mahler RF Hales CN. Improvement in insulin secretion in diabetes after diazoxide. Lancet (1976) 1:444–7. 10.1016/S0140-6736(76)91473-2 55717

[39] Weyer C Hanson RL Tataranni PA Bogardus C Pratley RE. A high fasting plasma insulin concentration predicts type 2 diabetes independent of insulin resistance: evidence for a pathogenic role of relative hyperinsulinemia. Diabetes (2000) 49:2094–101. 10.2337/diabetes.49.12.2094 11118012

[40] Standridge M Alemzadeh R Zemel M Koontz J Moustaid-Moussa N. Diazoxide down-regulates leptin and lipid metabolizing enzymes in adipose tissue of Zucker rats. FASEB J. (2000) 14:455–60. 10.1096/fasebj.14.3.455 10698960

[41] Alemzadeh R Fledelius C Bodvarsdottir T Sturis J. Attenuation of hyperinsulinemia by NN414, a SUR1/Kir6.2 selective K-adenosine triphosphate channel opener, improves glucose tolerance and lipid profile in obese Zucker rats. Metabolism (2004) 53:441–7. 10.1016/j.metabol.2003.10.027 15045689

[42] Bjorklund A Bondo Hansen J Falkmer S Grill V. Openers of ATP-dependent K+-channels protect against a signal-transduction-linked and not freely reversible defect of insulin secretion in a rat islet transplantation model of Type 2 diabetes. Diabetologia (2004) 47:885–91. 10.1007/s00125-004-1386-7 15088085

[43] Carr RD Brand CL Bodvarsdottir TB Hansen JB Sturis J. NN414, a SUR1/Kir6.2-selective potassium channel opener, reduces blood glucose and improves glucose tolerance in the VDF Zucker rat. Diabetes (2003) 52:2513–8. 10.2337/diabetes.52.10.2513 14514634

[44] Ritzel RA Hansen JB Veldhuis JD Butler PC. Induction of beta-cell rest by a Kir6.2/SUR1-selective K(ATP)-channel opener preserves beta-cell insulin stores and insulin secretion in human islets cultured at high (11 mM) glucose. J Clin Endocrinol Metab. (2004) 89:795–805. 10.1210/jc.2003-031120 14764798

[45] Zdravkovic M Kruse M Rost KL Moss J Kecskes A. The effects of NN414, a SUR1/Kir6.2 selective potassium channel opener in subjects with type 2 diabetes. Exp Clin Endocrinol Diabetes (2007) 115:405–6. 10.1055/s-2007-973062 17701889

[46] Zdravkovic M Kruse M Rost KL Moss J Kecskes A Dyrberg T. The effects of NN414, a SUR1/Kir6.2 selective potassium channel opener, in healthy male subjects. J Clin Pharmacol. (2005) 45:763–72. 10.1177/0091270005276947 15951466

[47] Fruchart JC Duriez P. Mode of action of fibrates in the regulation of triglyceride and HDL-cholesterol metabolism. Drugs Today (2006) 42:39–64. 10.1358/dot.2006.42.1.963528 16511610

[48] Alonso-Magdalena P Quesada I Nadal A. Endocrine disruptors in the etiology of type 2 diabetes mellitus. Nat Rev Endocrinol. (2011) 7:346–53. 10.1038/nrendo.2011.56 21467970

[49] Alonso-Magdalena P Ropero AB Soriano S Garcia-Arevalo M Ripoll C Fuentes E. Bisphenol-A acts as a potent estrogen via non-classical estrogen triggered pathways. Mol Cell Endocrinol. (2012) 355:201–7. 10.1016/j.mce.2011.12.012 22227557

[50] Persaud SJ Al-Majed H Raman A Jones PM. Gymnema sylvestre stimulates insulin release *in vitro* by increased membrane permeability. J Endocrinol. (1999) 163:207–12. 10.1677/joe.0.1630207 10556769

[51] Hoa NK Norberg A Sillard R Van Phan D Thuan ND Dzung DT. The possible mechanisms by which phanoside stimulates insulin secretion from rat islets. J Endocrinol. (2007) 192:389–94. 10.1677/joe.1.06948 17283239

[52] Szopa TM Ward T Dronfield DM Portwood ND Taylor KW. Coxsackie B4 viruses with the potential to damage beta cells of the islets are present in clinical isolates. Diabetologia (1990) 33:325–8. 10.1007/BF00404634 2165944

[53] Reed MA Pories WJ Chapman W Pender J Bowden R Barakat H. Roux-en-Y gastric bypass corrects hyperinsulinemia implications for the remission of type 2 diabetes. J Clin Endocrinol Metab. (2011) 96:2525–31. 10.1210/jc.2011-0165 21593117

[54] Teta M Long SY Wartschow LM Rankin MM Kushner JA. Very slow turnover of beta-cells in aged adult mice. Diabetes (2005) 54:2557–67. 10.2337/diabetes.54.9.2557 16123343

[55] Adams TD Davidson LE Litwin SE Kim J Kolotkin RL Nanjee MN. Weight and metabolic outcomes 12 years after gastric bypass. N Engl J Med. (2017) 377:1143–55. 10.1056/NEJMoa1700459 28930514 PMC5737957

[56] Dogan K Betzel B Homan J Aarts EO Ploeger N de Boer H. Long-term effects of laparoscopic Roux-en-Y gastric bypass on diabetes mellitus, hypertension and dyslipidaemia in morbidly obese patients. Obes Surg. (2014) 24:1835–42. 10.1007/s11695-014-1310-2 25027982

[57] Cummings DE Arterburn DE Westbrook EO Kuzma JN Stewart SD Chan CP. Gastric bypass surgery vs intensive lifestyle and medical intervention for type 2 diabetes: the CROSSROADS randomised controlled trial. Diabetologia (2016) 59:945–53. 10.1007/s00125-016-3903-x 26983924 PMC4826815

[58] Kulkarni AS Brutsaert EF Anghel V Zhang K Bloomgarden N Pollak M. Metformin regulates metabolic and nonmetabolic pathways in skeletal muscle and subcutaneous adipose tissues of older adults. Aging Cell (2018) 17:e12723. 10.1111/acel.12723 29383869 PMC5847877

[59] Retnakaran R Choi H Ye C Kramer CK Zinman B. Two-year trial of intermittent insulin therapy vs metformin for the preservation of beta-cell function after initial short-term intensive insulin induction in early type 2 diabetes. Diabetes Obes Metabol. (2018) 20:1399–407. 10.1111/dom.13236 29377408

[60] Patane G Piro S Rabuazzo AM Anello M Vigneri R Purrello F. Metformin restores insulin secretion altered by chronic exposure to free fatty acids or high glucose: a direct metformin effect on pancreatic beta-cells. Diabetes (2000) 49:735–40. 10.2337/diabetes.49.5.735 10905481

[61] Boden G Chen X Iqbal N. Acute lowering of plasma fatty acids lowers basal insulin secretion in diabetic and nondiabetic subjects. Diabetes (1998) 47:1609–12. 10.2337/diabetes.47.10.1609 9753299

[62] Boden G Chen X Rosner J Barton M. Effects of a 48-h fat infusion on insulin secretion and glucose utilization. Diabetes (1995) 44:1239–42. 10.2337/diab.44.10.1239 7556964

[63] Boden G Chen X Ruiz J White JV Rossetti L. Mechanisms of fatty acid-induced inhibition of glucose uptake. J Clin Invest. (1994) 93:2438–46. 10.1172/JCI117252 8200979 PMC294452

[64] Ader M Stefanovski D Kim SP Richey JM Ionut V Catalano KJ. Hepatic insulin clearance is the primary determinant of insulin sensitivity in the normal dog. Obesity (2014) 22:1238–45. 10.1002/oby.20625 24123967 PMC3969862

[65] Hsu IR Kim SP Kabir M Bergman RN. Metabolic syndrome, hyperinsulinemia, and cancer. Am J Clin Nutr. (2007) 86:s867–71. 10.1093/ajcn/86.3.867S 18265480

[66] Flory JH Ellenberg S Szapary PO Strom BL Hennessy S. Antidiabetic action of bezafibrate in a large observational database. Diabetes Care (2009) 32:547–51. 10.2337/dc08-1809 19131462 PMC2660490

[67] Tenenbaum A Fisman EZ. Balanced pan-PPAR activator bezafibrate in combination with statin: comprehensive lipids control and diabetes prevention? Cardiovasc Diabetol. (2012) 11:140. 10.1186/1475-2840-11-140 23150952 PMC3502168

[68] Tenenbaum A Fisman EZ. Fibrates are an essential part of modern anti-dyslipidemic arsenal: spotlight on atherogenic dyslipidemia and residual risk reduction. Cardiovasc Diabetol. (2012) 11:125. 10.1186/1475-2840-11-125 23057687 PMC3489608

[69] Tenenbaum H Behar S Boyko V Adler Y Fisman EZ Tanne D. Long-term effect of bezafibrate on pancreatic beta-cell function and insulin resistance in patients with diabetes. Atherosclerosis (2007) 194:265–71. 10.1016/j.atherosclerosis.2006.08.005 16970952

[70] Teramoto T Shirai K Daida H Yamada N. Effects of bezafibrate on lipid and glucose metabolism in dyslipidemic patients with diabetes: the J-BENEFIT study. Cardiovasc Diabetol. (2012) 11:29. 10.1186/1475-2840-11-29 22439599 PMC3342914

[71] Taylor KW Novak RF Anderson HA Birnbaum LS Blystone C Devito M. Evaluation of the association between persistent organic pollutants (POPs) and diabetes in epidemiological studies: a national toxicology program workshop review. Environ Health Perspect. (2013) 121:774–83. 10.1289/ehp.1205502 23651634 PMC3701910

[72] Kuo CC Moon K Thayer KA Navas-Acien A. Environmental chemicals and type 2 diabetes: an updated systematic review of the epidemiologic evidence. Curr Diab Rep. (2013) 13:831–49. 10.1007/s11892-013-0432-6 24114039 PMC4327889

[73] Behl M Rao D Aagaard K Davidson TL Levin ED Slotkin TA. Evaluation of the association between maternal smoking, childhood obesity, and metabolic disorders: a national toxicology program workshop review. Environ Health Perspect. (2013) 121:170–80. 10.1289/ehp.1205404 23232494 PMC3569686

[74] Zimmet PZ. Diabetes and its drivers: the largest epidemic in human history? Clin Diabetes Endocrinol. (2017) 3:1. 10.1186/s40842-016-0039-3 28702255 PMC5471716

